# Short-term associations between air pollution and nocturnal cough frequency

**DOI:** 10.1038/s44528-026-00030-5

**Published:** 2026-09-15

**Authors:** Tong Xia, Emil Carlsson, Mikael Kågebäck, Marco Montagna, John S. Ji, Cecilia Mascolo

**Affiliations:** 1 Vanke School of Public Health, Tsinghua University, Beijing, China; 2 Department of Computer Science and Technology, University of Cambridge, Cambridge, UK; 3 Sleep Cycle AB, Gothenburg, Sweden; 4 Department of Medicine, Vita-Salute San Raffaele University, Milan, Italy

**Keywords:** Climate sciences, Diseases, Environmental sciences

## Abstract

**Background:**

Air pollution remains a leading environmental risk factor for health, yet its short-term associations with early respiratory symptoms are not well characterized because population-scale symptom surveillance data are scarce. We aimed to quantify the association between short-term exposure to fine particulate matter (PM_2.5_) and nocturnal cough frequency using large-scale mobile sensing data collected across multiple countries.

**Methods:**

We analyzed nocturnal audio recordings from users of a widely used mobile sleep application (Sleep Cycle). An artificial intelligence-based cough detection model was applied to estimate nightly cough frequency. Data were aggregated at the city-day level across 32 cities in 12 countries over approximately 500 consecutive days. We used city-specific time-series models combined through random-effects meta-analysis and multi-city pooled models adjusting for meteorological conditions and influenza activity. In addition, we conducted an event-based analysis during a major wildfire episode to evaluate the acute respiratory impacts of extreme air pollution.

**Results:**

Higher daily PM_2.5_ concentrations were consistently associated with increased nocturnal cough frequency across all analytical approaches. In the city-specific meta-analysis, each 10 μg/m^3^ increase in PM_2.5_ was associated with a 2.4% increase in nocturnal cough frequency (relative risk 1.024, 95% CI: 1.013-1.035). Pooled analyses showed a nonlinear exposure-response relationship, with measurable increases in cough frequency observed even at relatively low PM_2.5_ concentrations. Elevated cough frequency was also observed during the wildfire episode, coinciding with substantial increases in ambient PM_2.5_.

**Conclusions:**

Short-term exposure to PM_2.5_ is associated with increased nocturnal cough frequency at the population level, suggesting that respiratory symptoms may respond to air pollution at lower exposure levels than previously recognized. Large-scale mobile sensing of respiratory symptoms provides a feasible digital biomarker for real-time environmental health surveillance and could complement traditional public health monitoring systems.

## Introduction

Air pollution is a leading global health threat, contributing to an estimated seven million premature deaths each year through cardiovascular and respiratory pathways^1^. Among airborne pollutants, fine particulate matter (PM_2.5_; aerodynamic diameter ≤2.5 μm) is of particular concern because these particles can penetrate deep into the lungs and enter the bloodstream, triggering systemic inflammation and cardiopulmonary dysfunction^2^.

While long-term exposure to PM_2.5_ is a well-established driver of cardiopulmonary morbidity and mortality^3,4^, the short-term respiratory effects of PM_2.5_ remain less clearly characterized. Time-series and case-crossover studies have reported acute increases in respiratory hospital admissions and reductions in lung function following short-term elevations in PM_2.5_ concentrations^5–7^. However, these studies predominantly capture severe clinical outcomes and rely on hospital-based records, thereby overlooking early and transient respiratory responses that occur at the population level. Moreover, most existing evidence is derived from single-city or regional studies (i.e., often in North America or East Asia) and typically relies on weekly or multi-day exposure averages rather than daily measurements.

Overall, existing studies suggest that short-term PM_2.5_ exposure may adversely affect respiratory health, yet the available evidence remains largely focused on severe clinical outcomes and geographically restricted settings. This limitation is increasingly consequential in the era of *health-oriented air pollution governance*, as reflected in the World Health Organization Air Quality Guidelines^1^, which emphasize reducing the overall population health burden rather than only preventing extreme clinical events^8^. A key evidence gap therefore remains: *whether short-term PM*_*2.5*_* exposure is associated with early, non-clinical respiratory symptoms across diverse real-world environments?* Addressing this question requires high-resolution, population-scale data capable of capturing subtle respiratory fluctuations with both spatial and temporal granularity, which are rarely available from conventional surveys or administrative health records.

Recent advances in mobile health (mHealth) technologies offer new opportunities to address this challenge. The widespread adoption of smartphones and wearable devices enables continuous, real-world health monitoring across large populations^9–11^. Among emerging sensing modalities, nocturnal audio monitoring provides a non-invasive and scalable method for detecting cough-a quantifiable marker of airway irritation and early respiratory dysfunction^12,13^. Modern AI algorithms can identify cough events from nighttime audio recordings with high accuracy, enabling large-scale and near-real-time assessment of respiratory activity^14–17^. Because nocturnal cough may reflect both environmental triggers and infection-related inflammation, it offers a unique window into early respiratory responses to air pollution.

Leveraging this opportunity, we analyzed 500 consecutive days of nighttime audio data collected through a widely used mobile sleep application (Sleep Cycle), spanning 32 cities in 12 countries across six continents and PM_2.5_ concentrations ranging from < 5 to > 40 μg/m^3^. An artificial intelligence algorithm identified cough events from raw audio recordings and aggregated them to the city-day level. Nocturnal cough frequency was calculated as cough events per recorded sleep hour across all users in each city-day. Linking these data with daily city-level PM_2.5_ concentrations enabled us to examine short-term associations between air pollution and nocturnal respiratory symptoms with fine temporal and spatial resolution.

Our analytical framework integrates three complementary perspectives. First, at the city level, we applied generalized additive models (GAMs) to estimate associations between daily PM_2.5_ concentrations and nocturnal cough frequency while flexibly adjusting for meteorological conditions and seasonal patterns. City-specific estimates were subsequently synthesized using random-effects meta-analysis to assess consistency across locations. Second, to characterize the overall exposure-response relationship, we fitted multi-city panel models with city and day fixed effects. This pooled model enables the evaluation of lagged and non-linear associations between PM_2.5_ and cough frequency with greater statistical power. Finally, we leveraged wildfire smoke episodes as quasi-natural experiments and applied an event-based interrupted time-series (ITS) framework to examine the short-term respiratory response to abrupt increases in PM_2.5_ concentrations.

In conclusion, this study demonstrates that short-term exposure to ambient PM_2.5_ is associated with increased nocturnal cough frequency. Across cities, a 10 μg/m^3^ increase in PM_2.5_ is associated with a 2.4% increase in nocturnal cough frequency (relative risk 1.024, 95% CI: 1.013–1.035), with evidence of a nonlinear exposure-response relationship and detectable associations below the WHO 24-h guideline value. Consistent findings from wildfire smoke episodes further support the robustness of this association. These results suggest that passively monitored nocturnal cough frequency may provide a scalable digital indicator for tracking the respiratory health impacts of air pollution.

## Methods

**Fig. 1:**
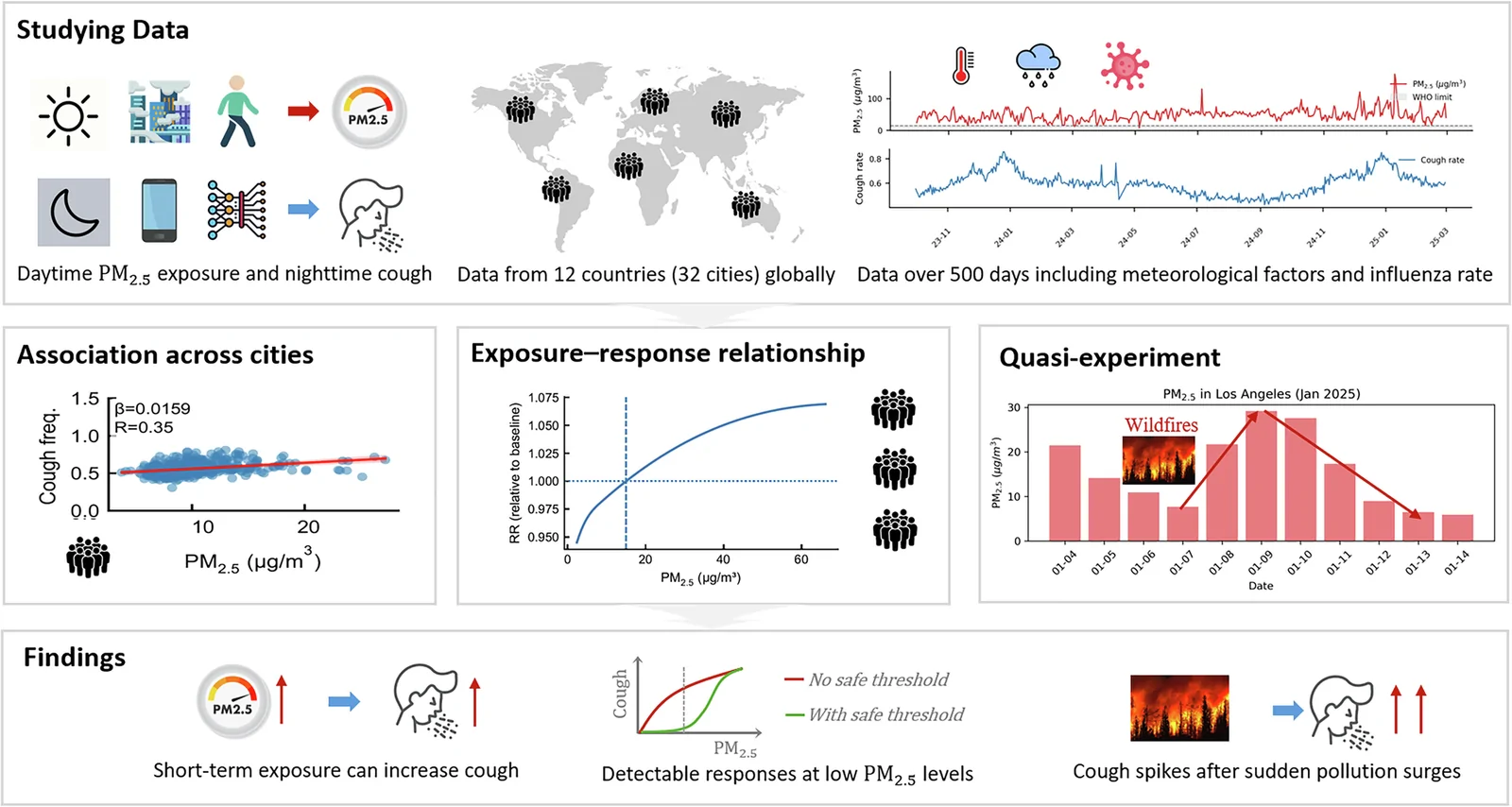
Study overview and analytical framework linking PM_2.5_ exposure to nocturnal cough frequency. Daily nocturnal cough events were passively detected from smartphone-based sleep recordings using an AI cough-detection algorithm and aggregated to the city-day level across 32 cities in 12 countries. These data were linked with city-level PM_2.5_ concentrations, meteorological variables, and influenza surveillance data over a 500-day observation period. The analytical framework integrates three complementary components: (i) within-city associations estimated using GAMs, (ii) multi-city pooled analyses characterizing the exposure-response relationship between PM_2.5_ and cough frequency, and (iii) ITS analyses examining respiratory responses during wildfire-related pollution surges. Together, these analyses evaluate population-level associations between short-term PM_2.5_ exposure and nocturnal cough frequency across diverse urban environments.

Our analytical framework integrates three complementary approaches, as shown in Fig. 1. First, we estimated city-specific associations between PM_2.5_ concentrations and nocturnal cough frequency using regression models and combined the estimates through random-effects meta-analysis, following a two-stage framework widely used in multi-city environmental epidemiology studies^18,19^. Second, we characterized the overall exposure-response relationship using pooled multi-city panel analyses. Third, we examined temporal associations between PM_2.5_ concentrations and nocturnal cough frequency during wildfire smoke episodes, which provided a natural setting for evaluating changes in cough frequency under rapidly varying air pollution conditions. Data sources and analytical methods are described below.

### Data sources

#### Cough data

Nighttime cough data were obtained from Sleep Cycle (Sleep Cycle AB, Sweden), a widely used mobile health application for sleep monitoring. The app passively records nighttime audio using the smartphone’s built-in microphone, typically between 10:00 PM and 7:00 AM. Cough events are automatically detected and timestamped using a validated machine learning algorithm that has demonstrated high precision (>90%) and recall (>85%) in previous evaluations^14,20^. The basic unit of observation is a sleep recording session, corresponding to a user’s nightly sleep recording. For each session, we extracted the total number of detected cough events and the recording duration. To account for variation in sleep duration across individuals and nights, nocturnal cough frequency for city *i* at day *t* was defined as:

$${{\mbox{Cough}}}_{it}=\frac{\sum {{\mbox{Cough}}}\, {{{\rm{Events}}}}_{it}}{\sum {{\mbox{Session}}}\, {{{\rm{Hours}}}}_{it}}.$$

This metric represents the number of cough events per hour of recorded sleep and provides a standardized measure that is robust to differences in recording duration. Daily cough rates were subsequently aggregated at the city-date level for analysis. High-resolution administrative boundaries from the Global Administrative Areas (GADM) database were used for geographic mapping. All data were collected as anonymized usage statistics and contained no personal identifiers. Optional demographic information (age and sex) was self-reported and analyzed only in aggregated form. Additional methodological details are provided in the following section.

**App usage and user distribution**. Because the dataset is aggregated at the city-day level rather than tracking a fixed cohort, we assessed user engagement using activity coverage metrics. Defining an active week as a week with at least one recorded sleep session, users contributed an average of 5.2 sessions per active week, corresponding to 72.3% weekly coverage. Similarly, users recorded an average of 18.1 sessions per active month, corresponding to 60.1% monthly coverage. These findings indicate substantial repeat participation and suggest that the dataset is not dominated by one-time recordings.

**Cough frequency distribution**. To characterize cough activity within the study population, we examined the distribution of average nocturnal cough frequency across users. As shown in Supplementary Fig. 1, the distribution was highly right-skewed, with the majority of users exhibiting very low cough frequencies during sleep. Specifically, 51.38% of users had no recorded cough events, 74% had fewer than 0.5 cough events per hour on average, and more than 90% had fewer than two cough events per hour. Only 0.83% of users exhibited more than 10 cough events per hour. This distribution is broadly consistent with previous objective nocturnal cough monitoring studies. For example, a controlled study comparing healthy subjects with patients with cystic fibrosis (CF) and primary ciliary dyskinesia (PCD) reported a median nocturnal cough frequency of 0.0 coughs/h among healthy participants, compared with approximately 1.3–2.3 coughs/h in CF and 0.2–0.5 coughs/h in PCD^21^. Consistent with these findings, the median nocturnal cough frequency in our dataset was also 0 coughs/h, suggesting that the study population was not substantially enriched for individuals with frequent nocturnal respiratory symptoms.

#### PM_2.5_ exposure data

Ambient PM_2.5_ data were obtained from the OpenAQ platform via its public application programming interface (API), which aggregates measurements from governmental monitoring networks worldwide. For each city, all monitoring stations with available PM_2.5_ measurements during the study period were identified. Stations with sufficient temporal coverage (i.e., data available for at least 500 days) were included to ensure reliable estimation of long-term exposure patterns. Daily city-level PM_2.5_ concentrations were calculated as the mean of station-level daily averages across all included stations. This approach reduces potential selection bias and provides a more representative estimate of ambient PM_2.5_ exposure within each city. All concentrations were reported in μg/m^3^. Days were included if at least one station reported valid measurements.

#### Other data

In addition to air pollution data, we incorporated meteorological variables-including daily mean temperature and precipitation-as model covariates. Because influenza infection is a major biomedical driver of cough^22^, influenza incidence data were obtained from national surveillance systems and converted into standardized city-level influenza rates.

**Meteorological data**. Daily meteorological variables, including average temperature (°F) and total precipitation (mm), were obtained from the U.S. National Oceanic and Atmospheric Administration Integrated Surface Dataset. For each study city, we selected the nearest meteorological station with complete data coverage during the study period (October 2023 to April 2025). Temperature values were converted from °F to °C prior to analysis.

**Influenza rate**. Data on influenza activity were obtained from official weekly surveillance reports published by national health agencies in each participating country, including the China National Influenza Center, the UK Health Security Agency, and the Italian National Institute of Health (Istituto Superiore di Sanità). These data included weekly influenza-like illness rates or virologically confirmed case counts, reported at the regional or national level. Weekly values were linearly interpolated to generate daily estimates, which were then standardized within each country to create a continuous influenza activity index used in regression models.

### City-specific regression

To estimate city-specific associations between ambient PM_2.5_ concentrations and nocturnal cough frequency, we fitted a GAM for each city *i*:

$$\log({{{\rm{Coug}}}}{{{\rm{h}}}}_{it})={\beta }_{0,i} + {\beta }_{1,i}\cdot {{{\rm{PM}}}}_{2.5,it}+{f}_{1,i}({{\mbox{FluRate}}}_{it})+{f}_{2,i}({{{\mbox{Temp}}}}_{it}) +{f}_{3,i}({{\mbox{Precip}}}_{it})+{\varepsilon }_{it},$$

where Cough_*it*_ denotes the average nocturnal cough frequency in city *i* on day *t*, and PM_2.5,*it*_ is the same-day ambient PM_2.5_ concentration. Smooth functions *f*_1,*i*_(⋅)–*f*_3,*i*_(⋅) represent non-linear effects of influenza activity, temperature, and precipitation, modeled with cubic regression splines. The intercept *β*_0,*i*_ captures city-specific baseline cough levels, and *ε*_*it*_ is the residual error term. As a supplementary analysis, we additionally fitted city-specific GAMs with a spline term for PM_2.5_ in a subset of cities to explore potential nonlinear exposure-response relationships.

For interpretability, the estimated coefficient *β*_1_ was exponentiated to yield the relative risk (RR) per 10 μg/m^3^ increase in PM_2.5_:

$${{{\rm{RR}}}}=\exp ({\beta }_{1}\cdot 10),\qquad {{{\rm{RR}}}}_{{{\rm{95 \% CI}}}}=\left[\exp \left(\left({\beta }_{1}-1.96\,{{{\rm{SE}}}}_{{\beta }_{1}}\right)\cdot 10\right), \exp \left(\left({\beta }_{1}+1.96\,{{{\rm{SE}}}}_{{\beta }_{1}}\right)\cdot 10\right)\right].$$

In addition, cluster-robust standard errors were computed at the city level to account for serial correlation. Models were estimated separately for the full year, non-flu season (April–October), and flu season (November–March) to capture seasonal variation.

### Multi-city pooled regression

To quantify the overall multi-city association while accounting for unobserved heterogeneity, we employed a fixed-effects (within) panel regression model. This approach controls for time-invariant city-level factors such as socioeconomic status, healthcare infrastructure, or baseline cough prevalence.

The baseline specification (**Model 1**) was:

$$\log ({{{\rm{Cough}}}}_{it})= \;	 {\beta }_{1}\cdot {{{\rm{PM}}}}_{2.5,it}+{\beta }_{2}\cdot {{{\rm{FluRate}}}}_{it}+{\beta }_{3}\cdot {{{\rm{Temp}}}}_{it}\\ 	 +{\beta }_{4}\cdot {{{\rm{Precip}}}}_{it}+{\alpha }_{i}+{\gamma }_{t}+{\varepsilon }_{it},$$

where *α*_*i*_ denotes city fixed effects, *γ*_*t*_ denotes date fixed effects, and *ε*_*it*_ is an idiosyncratic error term.

To examine delayed associations, we additionally fitted a distributed lag specification (**Model 2**):

$$\log ({{{\rm{Cough}}}}_{it})= 	 {\sum }_{l=0}^{5}{\beta }_{1l}\cdot {{{\rm{PM}}}}_{2.5,i,t-l}+{\beta }_{2}\cdot {{{\rm{FluRate}}}}_{it}+{\beta }_{3}\cdot {{{\rm{Temp}}}}_{it} \\ 	 +{\beta }_{4}\cdot {{{\rm{Precip}}}}_{it}+{\alpha }_{i}+{\gamma }_{t}+{\varepsilon }_{it},$$

where *PM*_2.5,*i*,*t*−*l*_ denotes ambient PM_2.5_ concentration at lag *l* days. In this specification, lag-specific coefficients were estimated simultaneously, allowing the association at each lag to be interpreted conditional on the other lag terms. The cumulative lag 0–5 association was obtained by summing the lag-specific coefficients.

To explore potential non-linear associations between PM_2.5_ exposure and nocturnal cough, we extended the fixed-effects framework by replacing the linear PM_2.5_ term with natural cubic **spline basis** functions. Specifically, we modeled:

$$\log ({{{\rm{Cough}}}}_{it})= 	 {\sum }_{k=1}^{K}{\gamma }_{k}\,{B}_{k}({{{\rm{PM}}}}_{2.5,it})+{\beta }_{2}\cdot {{{\rm{FluRate}}}}_{it}+{\beta }_{3}\cdot {{{\rm{Temp}}}}_{it}\\ 	 +{\beta }_{4}\cdot {{{\rm{Precip}}}}_{it}+{\alpha }_{i}+{\gamma }_{t}+{\varepsilon }_{it},$$

where *B*_*k*_(⋅) denotes the *k*th natural spline basis function (with *K* = 4 degrees of freedom), and *γ*_*k*_ are the corresponding coefficients. Knots were placed at empirical quantiles (10th, 50th, and 90th percentiles) of the observed PM_2.5_ distribution.

### Event-based interrupted time-series analysis of the Los Angeles wildfire

To examine the short-term respiratory impact of the January 2025 Los Angeles wildfire, we conducted an event-based ITS analysis using Los Angeles data only. Let PM_2.5,*t*_ and Cough_*t*_ denote the observed daily PM_2.5_ concentration and nocturnal cough frequency on day *t*, respectively. To account for seasonal background variation, we first constructed historical baselines from the corresponding week of year in 2024. Specifically, for each week *w*, we defined ${\overline{{{{\rm{PM}}}}}}_{2.5,w}^{\,{{{\rm{hist}}}}}$ and ${\overline{{{{\rm{Cough}}}}}}_{w}^{\,{{{\rm{hist}}}}}$ as the mean PM_2.5_ concentration and mean nocturnal cough frequency in Los Angeles during week *w* of 2024. We then defined the daily deviations in 2025 relative to these historical seasonal baselines as:

$$\Delta {{{{\rm{PM}}}}}_{2.5,t}={{{{\rm{PM}}}}}_{2.5,t}-{\overline{{{{\rm{PM}}}}}}_{2.5,w(t)}^{\,{{{\rm{hist}}}}},\,\Delta {{{{\rm{Cough}}}}}_{t}={{{{\rm{Cough}}}}}_{t}-{\overline{{{{\rm{Cough}}}}}}_{w(t)}^{\,{{{\rm{hist}}}}},$$

where *w*(*t*) denotes the week of year corresponding to day *t*. To assess whether the wildfire onset was associated with abrupt changes in these deviations, we fitted segmented linear ITS models of the form

$$\Delta {Y}_{t}={\beta }_{constant}+{\beta }_{time}\,{{{{\rm{Time}}}}}_{t}+{\beta }_{post}\,{{{{\rm{Post}}}}}_{t}+{\beta }_{after}\,{{{{\rm{TimeAfter}}}}}_{t}+{\varepsilon }_{t},$$

where Δ*Y*_*t*_ denotes either ΔPM_2.5,*t*_ or ΔCough_*t*_, Time_*t*_ is a continuous time index, Post_*t*_ is an indicator equal to 1 on and after the wildfire onset date (January 7, 2025) and 0 otherwise, and TimeAfter_*t*_ denotes the number of days since wildfire onset (set to 0 before the event). In this specification, *β*_*post*_ captures the immediate level change after wildfire onset, whereas *β*_*after*_ captures the post-event change in slope. Heteroskedasticity- and autocorrelation-consistent standard errors were estimated using a Newey-West correction. City-specific regression models were applied to both cough anomalies relative to the corresponding period in 2024 and the original cough frequency, with the latter analyzed on the log scale to estimate relative risks.

To further quantify the short-term association between wildfire-related PM_2.5_ anomalies and cough anomalies, we restricted the analysis to a narrow event window surrounding wildfire onset and fitted a distributed-lag regression:

$$\Delta {{{{\rm{Cough}}}}}_{t}=\alpha +{\sum }_{l=0}^{4}{\theta }_{l}\,\Delta {{{{\rm{PM}}}}}_{2.5,t-l}+{\eta }_{t},$$

where ΔPM_2.5,*t*−*l*_ denotes the deviation in PM_2.5_ concentration at lag *l* days. The cumulative short-term effect across lags 0–4 was defined as ${\theta }_{{{{\rm{cum}}}}}=\mathop{\sum }_{l = 0}^{4}{\theta }_{l}$. This quantity represents the total change in nocturnal cough frequency associated with a 1 μg/m^3^ increase in PM_2.5_ deviation distributed across the current day and the preceding 4 days.

### Ethics declarations

This study was approved by the Department of Computer Science and Technology, University of Cambridge Research Ethics Committee [application number #2434]. Sleep Cycle users provide informed consent through the application’s terms and privacy policy, which explain that data may be collected and used for research and service improvement purposes. No personally identifiable information was available to the researchers. Cough events were detected locally on users’ devices, and only aggregated, anonymized cough-frequency measures at the city-day level were used in this study. Consequently, no individual-level health information or audio recordings were accessed by the research team. The study was conducted in accordance with relevant ethical guidelines and regulations.

## Results

### Descriptive statistics of the data

We analyzed data collected over an 18-month period (October 2023 to April 2025) across 32 cities in 12 countries spanning Asia, Europe, North America, South America, Oceania, and Africa, representing regions with the highest user activity. Cities were included if their average user rate (daily active users per population) exceeded 0.5 per 10,000 residents, corresponding approximately to at least one active user per 10,000 inhabitants. Demographic characteristics of the included populations are summarized in Supplementary Table 1. The dataset includes users of both genders and a broad age distribution across cities with diverse socio-demographic contexts.

Table 1 summarizes city-level descriptive statistics. The study sites span a broad environmental gradient-from relatively low-pollution cities such as Paris (mean PM_2.5_ < 10 μg/m^3^) to highly polluted megacities such as Beijing (mean PM_2.5_ > 40 μg/m^3^) and include locations in both hemispheres (e.g., Melbourne, Australia). This geographic and climatic diversity provides a wide range of exposure conditions for evaluating the relationship between PM_2.5_ and respiratory symptoms.

**Table 1:** Descriptive statistics of the study population by city

Country	City	PM_2.5_ (μg/m^3^)	Days	Hours (h)	Users	Cough frequency	Temp. (°C)	Prec. (mm)
Australia	Brisbane	5.04	509	21801.53	3207.90	0.58	22.34	0.17
Australia	Melbourne	6.90	511	32035.59	4733.06	0.57	15.28	0.08
Australia	Sydney	5.14	497	33913.19	5077.72	0.58	18.86	0.10
Brazil	Sao Paulo	13.29	501	16205.57	2610.39	0.69	21.14	0.14
Chile	Santiago	27.85	502	3304.35	529.85	0.73	15.68	0.02
China	Beijing	40.97	487	8676.64	1361.63	0.56	10.71	0.06
China	Shanghai	33.48	386	11831.51	1823.13	0.56	15.97	0.11
France	Paris	8.67	499	23640.09	3594.38	0.64	11.98	0.09
Germany	Berlin	9.52	497	18642.77	2796.61	0.59	9.74	0.07
Germany	Leipzig	9.13	497	2110.56	316.64	0.63	9.87	0.07
Italy	Bergamo	14.70	290	808.74	122.65	0.69	13.36	0.15
Italy	Florence	11.90	402	1665.54	255.41	0.85	5.14	0.07
Italy	Milano	13.65	292	11405.31	1778.10	0.69	13.36	0.15
Italy	Monza and Brianza	12.35	289	960.34	145.09	0.87	13.36	0.15
Italy	Rome	12.97	423	6396.10	1003.14	0.77	16.20	0.07
Japan	Osaka	9.18	499	35809.11	6022.17	0.77	16.47	0.15
Japan	Tokyo	9.27	499	119307.82	19961.92	0.71	15.97	0.17
South Africa	City of Cape Town	18.37	451	2619.96	388.20	0.68	17.96	0.26
South Africa	City of Johannesburg	15.70	445	2312.30	351.26	0.73	19.05	0.25
Sweden	Uppsala	5.47	511	3782.19	542.99	0.75	5.03	0.05
United Kingdom	Birmingham	9.25	511	4966.65	754.76	0.72	10.23	0.10
United Kingdom	Cambridge	10.05	511	4289.14	615.95	0.68	13.36	0.11
United Kingdom	London	9.91	502	91261.88	13624.78	0.61	10.96	0.09
United Kingdom	Oxford	9.95	508	5090.97	726.90	0.71	9.99	0.09
United States	Chicago	9.38	511	26136.04	3944.73	0.58	8.47	0.08
United States	Columbus	8.33	497	15362.96	2353.17	0.67	17.31	0.39
United States	Los Angeles	10.56	510	52715.50	8002.03	0.60	15.43	0.03
United States	New York	7.59	511	33945.85	5248.07	0.46	12.97	0.12
United States	Phoenix	8.79	510	20510.87	3072.00	0.67	22.72	0.01
United States	Pittsburgh	9.59	511	5883.64	893.38	0.65	10.27	0.12
United States	Saint Louis	8.47	511	7093.24	1056.83	0.62	14.16	0.13
United States	Salt Lake	7.76	510	9476.04	1416.31	0.63	12.00	0.04

PM_2.5_ denotes the average daily concentration of PM_2.5_ over the study period. Days represents the number of city-day observations with complete data on nocturnal cough frequency, PM_2.5_, and covariates included in the analysis. Hours represents the average daily recorded hours per city, and Users is the average number of users who recorded data. cough frequency indicates the average daily cough frequency. Temp. (^∘^C) and Prec. (mm) represent the average temperature and precipitation, respectively, for each city.

A representative time-series visualization for Los Angeles (United States) is shown in Supplementary Fig. 2. Visual inspection indicates that cough frequency broadly follows seasonal influenza patterns, consistent with previous studies^23,24^. However, transient increases in cough frequency frequently coincide with short-term PM_2.5_ peaks, suggesting that acute air pollution episodes may contribute to day-to-day respiratory variability beyond the underlying seasonal trend.

### Positive associations between PM_2.5_ and nocturnal cough across cities

Table 1 highlights substantial intercity variability in both air quality (mean PM_2.5_ ranging from < 5 to > 40 μg/m^3^) and nocturnal cough frequency (0.53–0.85 events per recording hour). These differences likely reflect variations in socioeconomic conditions, energy use, baseline respiratory health, and other contextual factors. To account for this heterogeneity, we first conducted city-specific analyses.

**Fig. 2:**
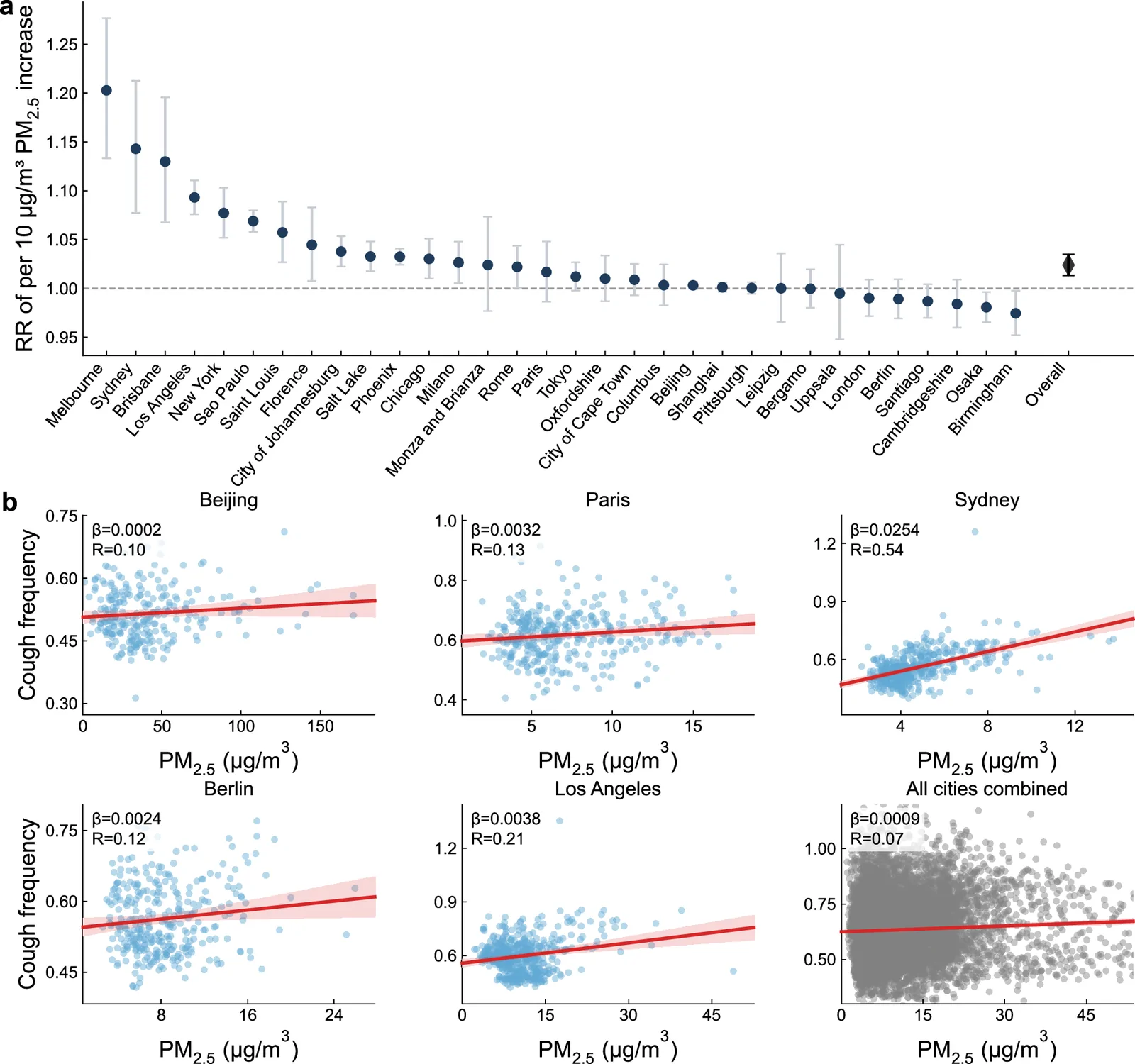
Associations between PM_2.5_ concentrations and nocturnal cough frequency across cities. **a** City-specific associations between same-day PM_2.5_ concentrations and nocturnal cough frequency estimated using GAMs with a linear PM_2.5_ term. Each city-specific estimate was based on 289–511 city-day observations, depending on data availability (Table 1). Each point represents the city-specific estimated RR, and error bars represent the corresponding 95% confidence intervals. **b** City-level scatter plots showing the relationship between PM_2.5_ concentrations and nocturnal cough frequency during non-flu periods (influenza rate ≤ 10%). Blue points represent individual city-day observations. The red line represents the fitted linear regression, and the shaded area denotes the corresponding 95% confidence interval of the fitted regression line. *β* denotes the estimated regression coefficient and *R* the Pearson correlation coefficient.

For each city, we fitted a GAM including a linear term for PM_2.5_ and spline terms for meteorological variables, temporal trend, and influenza activity to estimate short-term within-city associations. This specification yields a direct estimate of the relative risk (RR) associated with a 10 μg/m^3^ increase in PM_2.5_ for each city. The results are summarized in Fig. 2a, where cities are ordered by their estimated RR. Among the 32 cities, Melbourne (Australia) showed the largest estimated association (RR = 1.203, 95% CI: 1.133–1.278). Overall, 14 of the 32 cities had RR estimates greater than 1, suggesting that higher same-day PM_2.5_ concentrations were associated with increased nocturnal cough frequency in a subset of cities.

Building on the city-specific results, we next quantified the overall short-term association between PM_2.5_ and nocturnal cough while accounting for between-city variability. We conducted a random-effects meta-analysis using the DerSimonian-Laird method to pool city-level estimates and their standard errors^25^. The pooled effect estimate was 1.024 (95% CI: 1.013–1.035). Between-city heterogeneity was evident (*I*^2^ = 93.2%), although the estimated between-city variance was small (*τ*^2^ ≈ 0.00), indicating some variation in effect size across locations.

Because influenza epidemics may confound cough-related outcomes-individuals tend to cough more frequently during flu seasons regardless of air quality-we further examined the relationship during non-flu periods. The non-flu season was defined as days when the influenza rate remained below 10%. During these periods, influenza activity was consistently low across all cities, allowing a clearer assessment of the PM_2.5_-cough relationship. Positive linear relationships remained evident in 23 of the 32 cities. Some examples are given in Fig. 2b.

Although the above analyses used a linear PM_2.5_ term to obtain comparable RR estimates, we did not assume that the underlying exposure-response relationship was strictly linear. We therefore fitted more flexible spline-based models for a subset of cities with the strongest associations. As shown in Supplementary Fig. 3, the exposure-response curves varied across locations, indicating substantial heterogeneity in shape. Nevertheless, most cities exhibited increasing cough risk at higher PM_2.5_ concentrations, supporting the positive associations observed in the main analysis.

### Population-level exposure-response relationship between PM_2.5_ and cough

To further investigate temporal lag patterns and potential nonlinear exposure-response relationships, we pooled data from all cities to fit two-way fixed-effects panel regression models adjusting for meteorological variables and influenza activity. To maintain consistency with the city-specific analyses, effect estimates were expressed as RRs per 10 *μ*g/m^3^ increase in PM_2.5_.

**Fig. 3:**
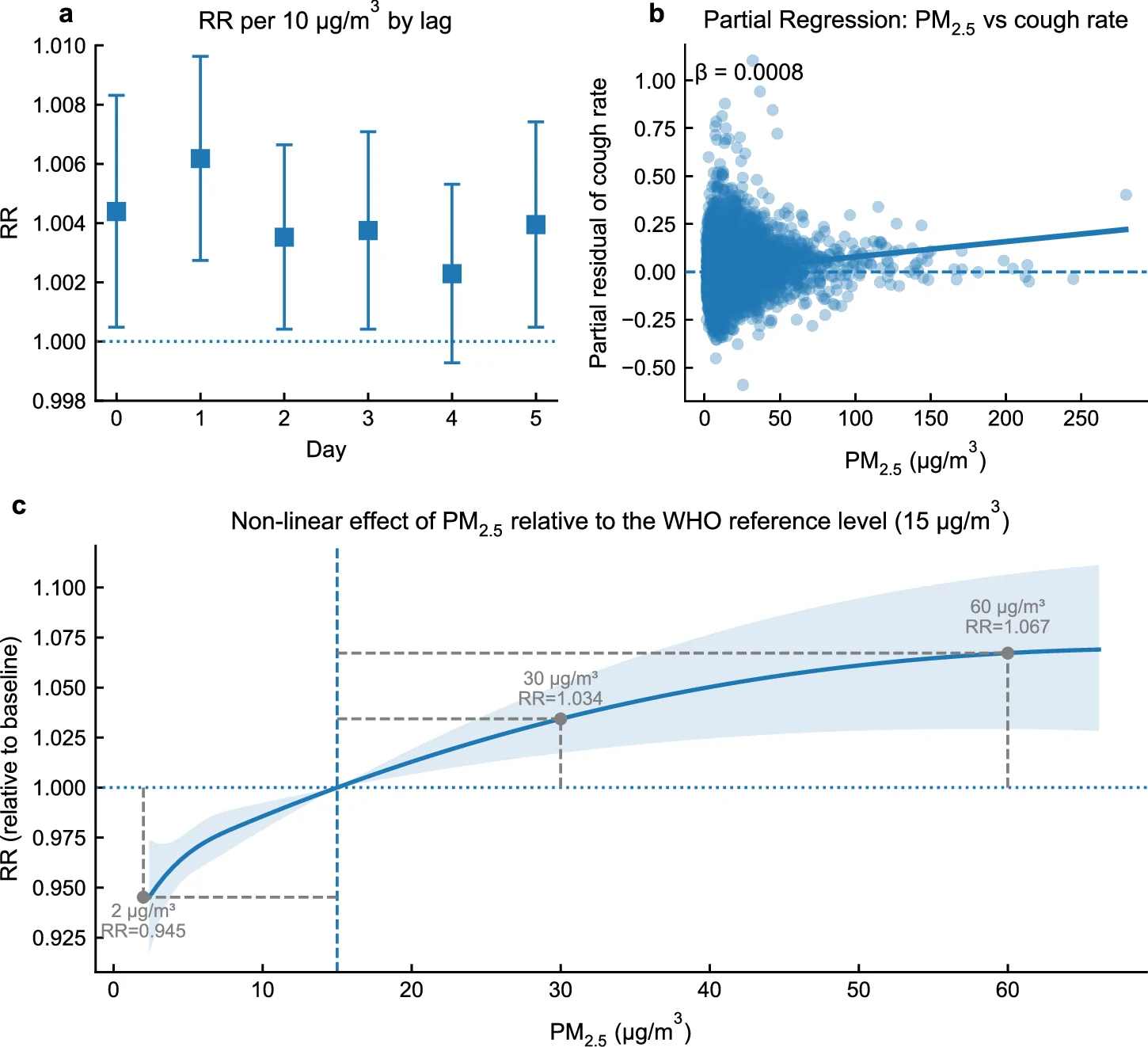
Population-level association between PM_2.5_ exposure and nocturnal cough frequency. **a** Lag-specific RRs of nocturnal cough per 10 μg/m^3^ increase in PM_2.5_ estimated from distributed lag models using 14896 city-day observations across 32 cities. Squares indicate point estimates, and error bars represent the corresponding 95% confidence intervals. **b** Partial regression analysis showing the relationship between PM_2.5_ (lag 0) and cough rate residuals, controlling for other factors; **c** Non-linear exposure-response curve for PM_2.5_ (lag 0) and cough frequency, where the solid line represents the fitted RR estimated from the spline model, and the shaded band represents the corresponding 95% confidence interval.

Table 2 summarizes the regression results from two model specifications: a single-lag model including only same-day exposure (lag 0; Model 1) and a distributed lag model including lags 0–5 simultaneously (Model 2). Short-term PM_2.5_ exposure was positively associated with nocturnal cough frequency in both specifications. In the single-lag model (Model 1), same-day PM_2.5_ was significantly associated with higher nocturnal cough frequency (*β* = 0.0012, *p* = 0.0002), corresponding to an RR of 1.012 (95% CI: 1.006–1.018) per 10 μg/m^3^ increase. In the distributed lag model (Model 2), the cumulative association across lags 0–5 remained statistically significant (*β* = 0.0024, *p* < 0.0001), corresponding to an RR of 1.024 (95% CI: 1.012–1.037) per 10 μg/m^3^ increase. Lag-specific RR estimates from the distributed lag model are shown in Fig. 3a. Point estimates of the RR per 10 μg/m^3^ increase in PM_2.5_ were above 1 across lag days 0–5, with the largest estimate at lag 1.

**Table 2:** Fixed-effects panel regression estimates of the association between PM_2.5_ exposure and nocturnal cough frequency

Variable	Estimate	Std. Error	95% CI	RR (per 10 μg/m^3^)	*P*-value
**Model 1: main results for Lag 0**					
PM_2.5_ (Lag 0)	0.0012	0.0003	[0.0006, 0.0018]	1.012 (1.006–1.018)	0.0002
Influenza rate	0.3669	0.0865	[0.1974, 0.5365]	–	0.0000
Temperature (°C)	−0.0110	0.0013	[−0.0136, −0.0084]	–	0.0000
Precipitation (mm)	0.0048	0.0054	[−0.0058, 0.0153]	–	0.8827
**Model 2: main results for distributed Lag model (Lag 0-5)**					
PM_2.5_ (Lag 0–5)	0.0024	0.0006	[0.0012, 0.0036]	1.024 [1.012, 1.037]	0.0001
Influenza rate	0.3636	0.0856	[0.1941, 0.5298]	–	0.0001
Temperature (°C)	−0.0069	0.0007	[−0.0074, −0.0045]	–	0.0000
Precipitation (mm)	0.0049	0.0053	[−0.0054, 0.0153]	–	0.3501

Estimates are from two-way fixed effects models (city and date fixed effects). Standard errors are clustered by both city and date. RR values represent the relative risk associated with a 10 μg/m^3^ increase in PM_2.5_. *P* values are derived from two-sided *t*-tests based on two-way cluster-robust standard errors (clustered by city and date) in the fixed-effects panel regression models.

Influenza activity was strongly associated with increased cough frequency in both models (*β* = 0.3669 and 0.3619, respectively; both *p* < 0.0001), with higher cough frequency observed during periods of elevated influenza activity. Sensitivity analyses further examined the potential role of influenza activity in shaping the observed PM_2.5_-cough association (Supplementary Table 2). The estimated PM_2.5_ effect remained similar after removing influenza adjustment or introducing an interaction term between PM_2.5_ and influenza incidence, indicating that the association between PM_2.5_ exposure and nocturnal cough frequency was robust to alternative model specifications. The interaction term was not statistically significant, suggesting limited evidence that influenza activity substantially modified the PM_2.5_ effect. Among meteorological covariates, higher ambient temperature was inversely associated with cough frequency (Model 1: *β* = −0.0061, 95% CI: [−0.0075, −0.0047]; Model 2: identical estimates), whereas precipitation was not significantly associated with cough in either model. Figure 3b presents the partial regression relationship between PM_2.5_ and cough frequency after adjusting for meteorological and influenza covariates. The positive slope (*β* = 0.0008) is consistent with the direction of most city-specific estimates and indicates a positive population-level association.

As shown in Fig. 3c, the exposure-response relationship between PM_2.5_ and the RR of cough was characterized by a pronounced non-linear pattern. Using the WHO 24-h air quality guideline (15 μg/m^3^) as the reference level (*RR* = 1.000), we observed that the risk of cough increased most sharply at the lower end of the exposure spectrum. Specifically, as PM_2.5_ concentrations decreased from the WHO guideline to a near-zero level (2 μg/m^3^), the RR significantly dropped to 0.945 (95% CI: 0.917–0.974), indicating a continuous health benefit from further air quality improvement even below current global standards. Conversely, when PM_2.5_ rose to 30 μg/m^3^-twice the WHO limit-the RR increased to 1.035 (95% CI: 1.017–1.052), representing a 3.4% excess risk. Notably, the curve exhibited a saturation effect at higher concentrations. While the RR continued to rise to 1.067 (95% CI: 1.029–1.106) at 60 μg/m^3^, the slope was markedly shallower than that observed in the 0–30 μg/m^3^ range. This steep slope at low-to-moderate concentrations underscores that the human respiratory system is exquisitely sensitive to PM_2.5_ fluctuations in relatively clean environments, suggesting no evident safety threshold for PM_2.5_-induced cough reflexes.

### Wildfire smoke episodes reveal short-term increases in cough frequency

**Fig. 4:**
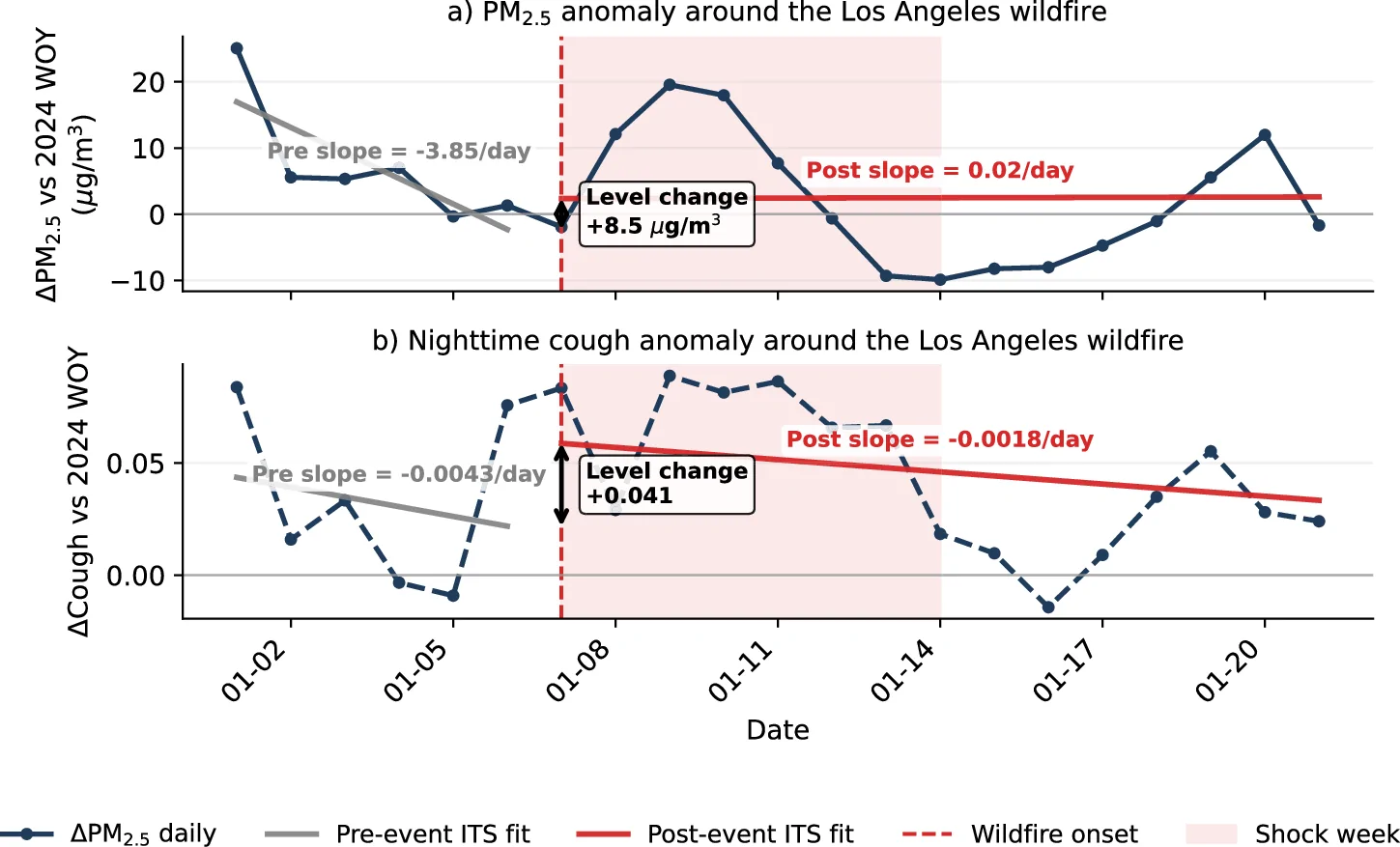
Quasi-experiment analysis of the Los Angeles wildfire episode. **a** PM_2.5_ anomalies and **b** nocturnal cough anomalies during the 2025 Los Angeles wildfire, expressed relative to the corresponding week of year in 2024. The dashed vertical line marks wildfire onset, and the shaded region indicates the subsequent smoke period. The annotated *level change* corresponds to the immediate post-wildfire shift *β*_*post*_, whereas the *pre slope* and *post slope* correspond to the estimated temporal trends before *β*_*time*_ and after *β*_*time*_ + *β*_*after*_ wildfire onset, respectively.

Using the event-based ITS framework described in Methods, we compared deviations in PM_2.5_ and nocturnal cough frequency relative to their historical seasonal baselines within a  ± 20-day window surrounding wildfire onset. As shown in Fig. 4, wildfire onset was associated with an immediate increase in PM_2.5_ of 8.52 μg/m^3^ (*β*_*post*_ = 8.52, *p* = 0.005), indicating a substantial perturbation in ambient air quality. Nocturnal cough frequency increased concurrently, with an estimated level change of 0.041 relative to baseline (*β*_*post*_ = 0.041, *p* = 0.056). Although the latter estimate was only borderline significant, the temporal alignment between the pollution spike and the increase in cough frequency suggests a rapid respiratory response to wildfire-related air pollution.

We then applied the distributed-lag model within the wildfire event window. Positive associations were observed at both lag 0 and lag 4 days (full results are summarized in Supplementary Table 3), indicating both immediate and delayed respiratory responses to PM_2.5_ exposure. The cumulative lag 0–4 day effect was 0.0056 per 1 μg/m^3^ increase in PM_2.5_ anomaly (95% CI: 0.0045–0.0068, *p* < 0.001), corresponding to an increase of 0.056 in nocturnal cough frequency per 10 μg/m^3^ increase in PM_2.5_. When modeled on the log scale of original cough frequency and PM_2.5_ concentration, a 10 μg/m^3^ increase in PM_2.5_ was associated with a 9.9% increase in nocturnal cough frequency. Notably, the wildfire-specific effect estimate exceeded the average association observed across the full multi-city dataset, consistent with the nonlinear exposure-response relationship identified in the primary analyses. Together, these findings suggest that extreme short-term PM_2.5_ exposures may produce disproportionately large increases in respiratory symptoms.

## Discussion

This study provides population-scale evidence that higher short-term PM_2.5_ concentrations are associated with higher nocturnal cough frequency, with associations detectable both on the day of exposure and the following day. The association was observed across multiple cities and remained robust after adjustment for meteorological conditions and influenza activity. These findings suggest that even relatively low concentrations of PM_2.5_ commonly encountered in urban environments may contribute to measurable respiratory symptoms, highlighting nocturnal cough as a sensitive indicator of airway irritation. The monotonic exposure-response relationship further suggests that respiratory responses may occur even at relatively low PM_2.5_ concentrations, underscoring the pervasive health burden associated with urban air pollution.

In our primary model, the estimated association corresponded to an RR of 1.012 (95% CI: 1.006–1.018) per 10 μg/m^3^ increase in PM_2.5_. This magnitude is consistent with effect sizes reported in previous air pollution epidemiology studies for short-term health outcomes. For example, large multi-city time-series analyses have reported increases of approximately 0.5–0.7% in daily mortality per 10 μg/m^3^ increase in PM_2.5_^26,27^. Because coughing represents a frequent symptomatic response rather than a rare endpoint such as mortality, a relative increase of approximately 1.2% per 10 μg/m^3^ is epidemiologically plausible and meaningful at the population level.

An important contribution of this work lies in the use of mobile health (mHealth) technologies for environmental epidemiology. By capturing nocturnal cough through smartphone-based monitoring combined with machine learning algorithms, we introduce a scalable, objective, and non-invasive approach for quantifying early respiratory responses in real-world settings. This approach complements conventional endpoints such as hospital admissions and mortality^28–32^. Our findings suggest that the health burden of air pollution may be underestimated when analyses focus solely on severe clinical outcomes. More broadly, these results highlight the potential of mHealth platforms to enable continuous, high-resolution monitoring of environmental health impacts across populations^10,11^.

Beyond its respiratory relevance, nocturnal cough may also have broader systemic implications. Sleep fragmentation caused by nighttime coughing could link air pollution exposure to cardiometabolic dysfunction, impaired cognitive performance, and neurodegenerative risk^33,34^. Thus, associations between short-term particulate exposure and nocturnal cough may be relevant to health outcomes extending beyond respiratory symptoms alone. This perspective shifts the focus of air pollution research from overt morbidity and mortality toward earlier manifestations that may affect quality of life and long-term well-being.

The wildfire case study provides complementary quasi-experimental evidence that abrupt increases in PM_2.5_ may be accompanied by short-term increases in respiratory symptoms beyond expected seasonal baselines. Although nocturnal cough frequency appeared to increase before the most pronounced rise in PM_2.5_, this early fluctuation may reflect background respiratory variability associated with factors other than wildfire-related air pollution, including seasonal respiratory infections or other unmeasured environmental exposures. Consequently, the wildfire analysis should be interpreted as complementary evidence supporting the short-term association between PM_2.5_ and nocturnal cough rather than definitive causal evidence that the wildfire itself initiated the observed increase in cough frequency. Nevertheless, the temporal co-occurrence of elevated PM_2.5_ and cough frequency, together with the distributed-lag analysis conducted within the wildfire period, supports a rapid respiratory response to severe air pollution episodes. These findings align with recent studies reporting increased hospitalizations during wildfire smoke events^35,36^. As climate change accelerates the frequency and intensity of wildfires and other extreme events^37–39^, their contribution to acute respiratory morbidity is likely to increase. Integrating climate resilience and early-warning systems into air quality management will therefore be essential to protect public health.

Several limitations should be considered. First, our exposure assessment relied on ambient PM_2.5_ concentrations measured at outdoor monitoring stations, whereas nocturnal cough occurs primarily in indoor environments. Indoor exposure levels may differ substantially from outdoor concentrations due to building characteristics, ventilation, and occupant behavior. The indoor-to-outdoor ratio of PM_2.5_ can vary widely across settings and may be affected by factors such as air conditioning, filtration systems, and the use of portable air purifiers. These factors may introduce exposure misclassification and attenuate effect estimates. Future studies incorporating personal or indoor air quality measurements would help refine exposure assessment and better characterize the relationship between indoor pollution and respiratory symptoms.

Second, the study population consisted of users of a mobile sleep monitoring application, with demographic characteristics (e.g., age distribution and sex ratio) and health-related behaviors that may differ from the general population, potentially limiting the generalizability of the findings. However, given that the prevalence of chronic inflammatory airway conditions increases with age and that such individuals are more susceptible to airborne environmental stressors, it is plausible that the observed effect size of short-term PM_2.5_ exposure on cough may be larger in older or clinically vulnerable subgroups.

Third, this study focused on PM_2.5_ and did not evaluate the potential contribution of coarse particulate matter (e.g., PM_10_) or other air pollutants. Because larger particles are more likely to deposit in the upper airways, where cough receptors are concentrated, their associations with nocturnal cough may differ from those observed for PM_2.5_. Future studies incorporating multiple pollutants could help disentangle their relative contributions to respiratory symptom patterns.

Finally, cough reflex sensitivity is known to be reduced during sleep, particularly during deeper sleep stages. As a result, nocturnal cough frequency may underestimate the overall burden of respiratory irritation associated with air pollution exposure. Furthermore, sleep-stage-specific suppression of cough could contribute additional variability that was not captured in the present analysis. Future studies integrating physiological sleep measurements may help clarify how sleep processes modify associations between air pollution and nocturnal cough.

In conclusion, our findings provide large-scale real-world evidence that short-term increases in PM_2.5_ are associated with higher nocturnal cough frequency across multiple cities. By leveraging mHealth data streams, this study demonstrates a scalable framework for monitoring early respiratory responses to air pollution in near real time. Integrating digital health data with environmental monitoring may therefore provide valuable opportunities to strengthen public health surveillance and inform air quality policy in an era of rapid urbanization and climate change.

## Supplementary information

Supplementary Information

## Data Availability

Nighttime cough data used in this study were obtained from the Sleep Cycle mobile health platform and are subject to privacy restrictions. For research purposes, de-identified aggregated data may be made available upon request to the corresponding author, subject to approval by the data provider. All other datasets, including daily PM$_{2.5}$ measurements (https://openaq.org/), meteorological records (https://www.ncei.noaa.gov/cdo-web/search), and influenza surveillance statistics (https://www.who.int/tools/flunet), are publicly accessible from official repositories as described in the “Methods” section.

## Code availability

The analyses were conducted in Python 3.9 using standard packages including pandas, geopandas, numpy, scipy, matplotlib, seaborn, and *linearmodels* for regressions. The code used for data processing, panel regression, and figure generation, along with the necessary data, is openly available from https://figshare.com/articles/online_resource/Repo/32668803.
