# Supplementary material for: Short-term associations between air pollution and nocturnal cough frequency
Source: Commun Health. 2026 Sep 15;1(1):27. doi: 10.1038/s44528-026-00030-5 (PMC13577914; doi:10.1038/s44528-026-00030-5)
Supplement: Supplementary file 1 [file 44528_2026_30_MOESM1_ESM.pdf]

Supplementary Information

Supplementary Table 1. Descriptive statistics of the study population by country. Values are reported as male-to-female ratio, mean age with standard deviation, and the 25th (Q1) and 75th (Q3) age percentiles.

Country	Male/Female Ratio	Age (Mean± Std)	Age Q1 (25%)	Age Q3 (75%)
Australia	0.93	34.9 ± 12.07	25	44
Brazil	1.74	36.9 ± 11.06	29	44
Chile	2.06	37.7 ± 12.08	28	47
China	1.45	32.6 ± 9.07	27	37
France	1.45	35.9 ± 13.02	25	47
Germany	1.87	36.7 ± 13.04	26	48
Italy	1.93	39.7 ± 13.05	28	49
Japan	1.83	43.0 ± 13.07	32	54
South Africa	2.00	41.76 ± 14.60	31	52
Sweden	1.14	38.31 ± 14.91	25	52
United Kingdom	1.23	37.0 ± 13.06	26	46
United States	1.07	39.8 ± 15.00	27	50

Notes: Demographic information were self-reported and from a subset of the app users that has given us consent to use their data for research. To protect user privacy, we do not report demographic information at the city level; instead, we provide aggregated country-level summaries.

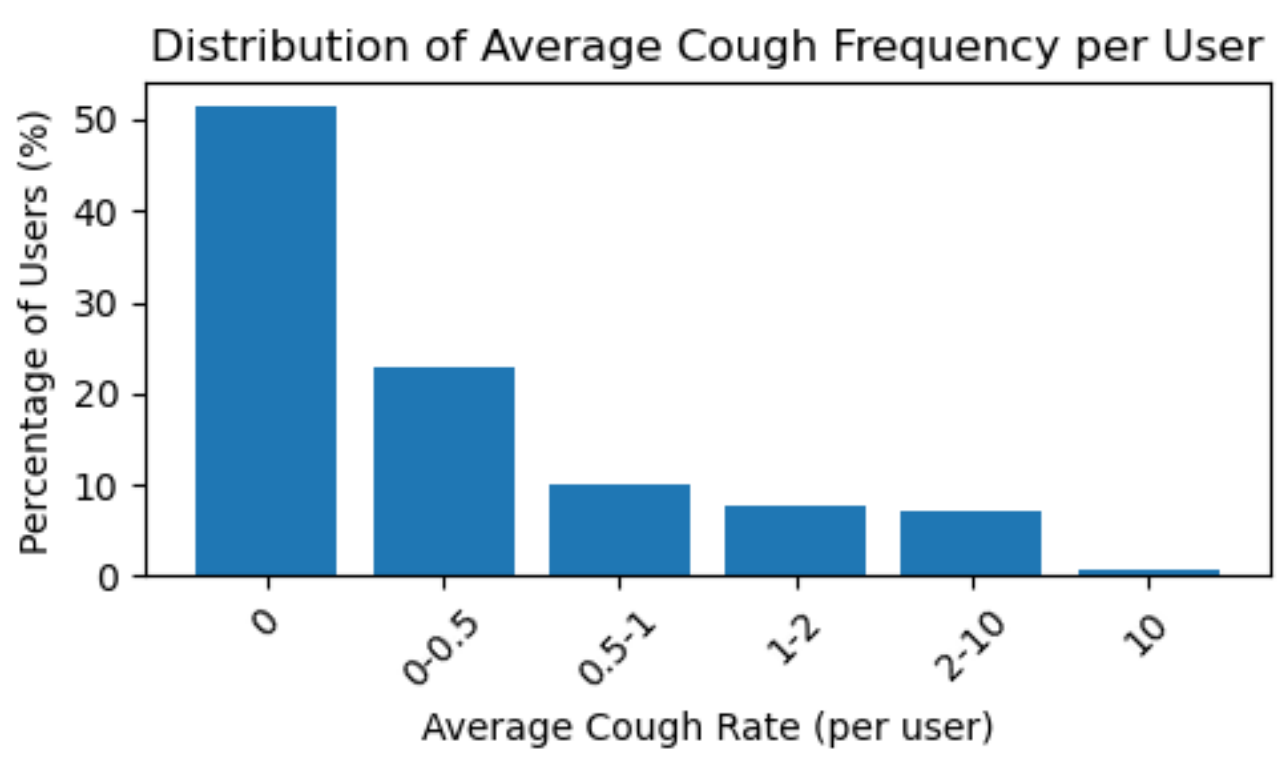

Supplementary Figure 1. Distribution of average nocturnal cough frequency per user. The majority of users exhibited little or no coughing during sleep, with over half of users having zero cough events overnight and more than 90% experiencing fewer than two cough events per hour.

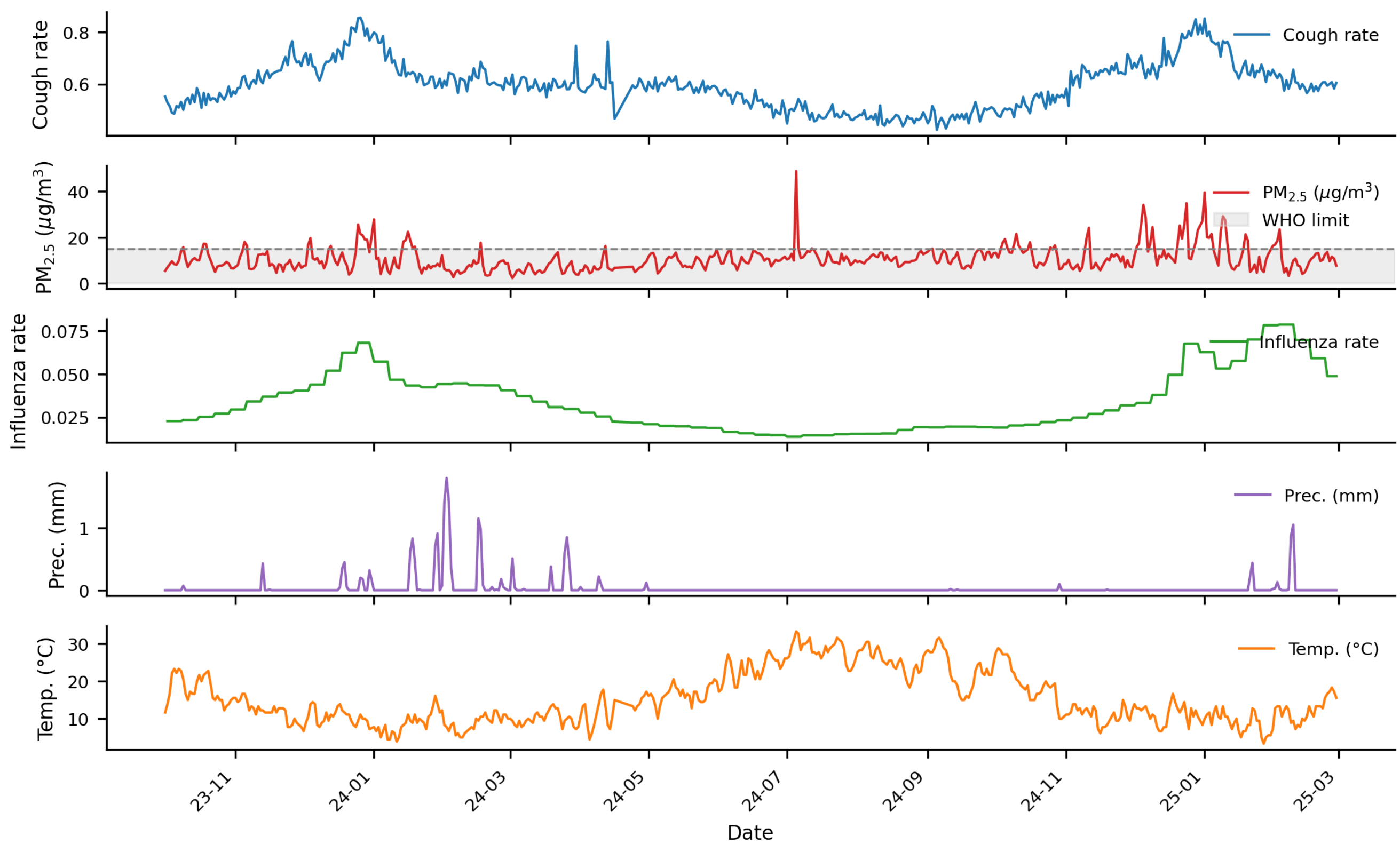

Supplementary Figure 2. Time series of health and environmental variables in Log Angeles over the 500-day study period. The panels display daily trends in cough rate, $PM_{2.5}$ concentration, influenza rate, precipitation, and temperature. The shaded region in the $PM_{2.5}$ panel indicates the WHO recommended exposure limit of 15 μg/m^3.

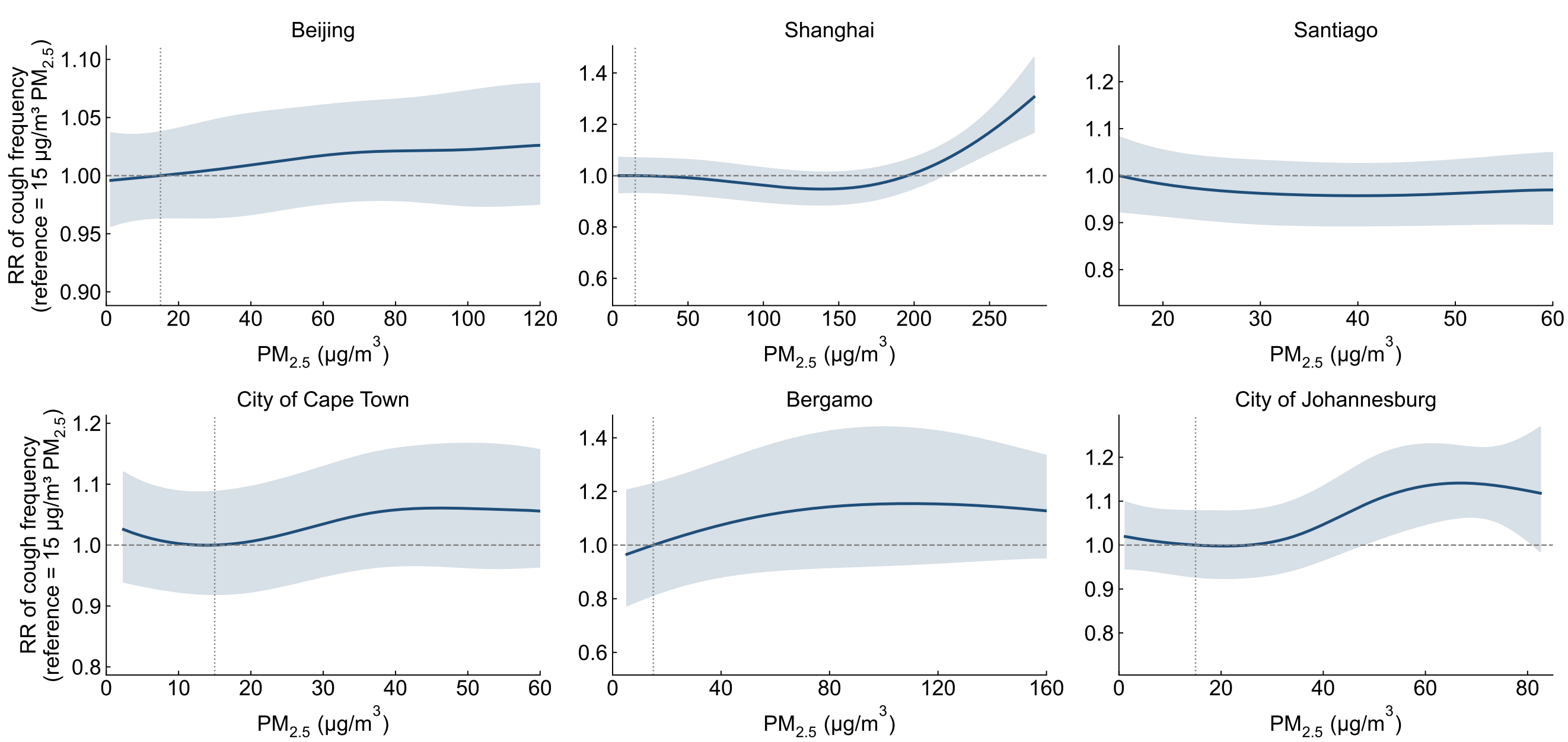

Supplementary Figure 3. City-specific nonlinear exposure-response relationships between $PM_{2.5}$ and cough frequency in cities with average $PM_{2.5}$ levels greater than 15 μg/m^3. Relative risks (RRs) were estimated from spline-based generalized additive models, with 15 μg/m^3 used as the reference concentration. Solid lines represent estimated RRs and shaded areas represent 95% confidence intervals. Models were adjusted for temporal trend, temperature, precipitation, and influenza activity.

Supplementary Table 2. Sensitivity analyses examining the role of influenza incidence in the association between $PM_{2.5}$ and nocturnal cough.

Variable	Estimate	Std. Error	95% CI	RR (per 10 $\mu g/m^3$)	P-value
**Model 1: Main Results for Lag 0**					
$PM_{2.5}$	0.0012	0.0003	[0.0006, 0.0018]	1.012 (1.006–1.018)	0.0002
Influenza rate	0.3669	0.0865	[0.1974, 0.5365]	–	< 0.0001
Temperature (°C)	-0.0110	0.0013	[-0.0136, -0.0084]	–	< 0.0001
Precipitation (mm)	0.0048	0.0054	[-0.0058, 0.0153]	–	0.3774
**Model 3: $PM_{2.5}$ (Lag 0) × Influenza Interaction**					
$PM_{2.5}$	0.0015	0.0004	[0.0007, 0.0022]	1.015 (1.007–1.022)	0.0001
Influenza rate	0.4315	0.1136	[0.2087, 0.6542]	–	0.0001
Temperature (°C)	-0.0110	0.0013	[-0.0135, -0.0085]	–	< 0.0001
Precipitation (mm)	0.0050	0.0053	[-0.0054, 0.0155]	–	0.3446
$PM_{2.5}$ × Influenza	-0.0039	0.0026	[-0.0090, 0.0012]	–	0.1372
**Model 4: $PM_{2.5}$ (Lag 0) and removing influenza rate**					
$PM_{2.5}$	0.0016	0.0004	[0.0008, 0.0024]	1.016 (1.008–1.024)	0.0001
Temperature (°C)	-0.0123	0.0016	[-0.0155, -0.0092]	–	< 0.0001
Precipitation (mm)	0.0024	0.0058	[-0.0090, 0.0138]	–	0.6792

Notes: Estimates are from two-way fixed-effects panel regression models with city and date fixed effects. Standard errors are clustered by both city and date. Relative risks (RRs) represent the multiplicative change in nocturnal cough frequency associated with a 10 $\mu g/m^3$ increase in $PM_{2.5}$, calculated as $\exp(10\beta)$. P values were derived from two-sided t-tests based on two-way cluster-robust standard errors. No adjustment for multiple comparisons was applied because $PM_{2.5}$ was the primary exposure specified *a priori*.

Supplementary Table 3. Distributed-lag regression analyses during the Los Angeles wildfire episode (event window: ±10 days around wildfire onset).

Predictor	Estimate	Std. Error	95% CI	P-value
**Panel A. Outcome: Δ nocturnal cough frequency**				
$PM_{2.5}$ lag 0	0.0020	0.0004	(0.0012, 0.0027)	<0.001
$PM_{2.5}$ lag 1	−0.0000	0.0010	(−0.0019, 0.0019)	0.983
$PM_{2.5}$ lag 2	−0.0011	0.0007	(−0.0024, 0.0002)	0.088
$PM_{2.5}$ lag 3	0.0047	0.0010	(0.0026, 0.0067)	<0.001
$PM_{2.5}$ lag 4	−0.0022	0.0007	(−0.0036, −0.0008)	0.002
Model R^2			0.735	
Observations			17	
**Panel B. Outcome: log(cough rate)**				
$PM_{2.5}$ lag 0	0.0047	0.0012	(0.0024, 0.0071)	<0.001
$PM_{2.5}$ lag 1	0.0009	0.0028	(−0.0047, 0.0064)	0.751
$PM_{2.5}$ lag 2	−0.0001	0.0019	(−0.0038, 0.0036)	0.956
$PM_{2.5}$ lag 3	0.0040	0.0015	(0.0011, 0.0069)	0.006
$PM_{2.5}$ lag 4	0.0016	0.0010	(−0.0004, 0.0036)	0.120
Model R^2			0.655	
Observations			17	

Notes: Models were estimated using heteroscedasticity- and autocorrelation-consistent (HAC) standard errors. P values were derived from two-sided t-tests based on two-way cluster-robust standard errors. No adjustment for multiple comparisons was applied. For Panel A, the cumulative lag 0–4 effect was 0.0056 per 1 $\mu g/m^3$ increase in $PM_{2.5}$ anomaly (95% CI 0.0045–0.0068; $p < 0.001$), corresponding to an increase of 0.056 in nocturnal cough frequency per 10 $\mu g/m^3$ increase in $PM_{2.5}$. For Panel B, the cumulative lag 0–4 relative risk associated with a 10 $\mu g/m^3$ increase in $PM_{2.5}$ anomaly was 1.099 (95% CI 1.081–1.117; $p < 0.001$), equivalent to a 9.9% increase in nocturnal cough frequency.
